# Farmers’ perceptions on stock theft in some districts of the Eastern Cape Province, South Africa

**DOI:** 10.1371/journal.pone.0310881

**Published:** 2024-09-27

**Authors:** Kanya Ndzungu, Ishmael Festus Jaja

**Affiliations:** 1 Department of Livestock and Pasture Science, University of Fort Hare, Alice, South Africa; 2 Department of Agriculture and Animal Health, University of South Africa, Roodepoort, Johannesburg, South Africa; Covenant University, NIGERIA

## Abstract

Stock theft is a persistent and widespread problem affecting farmers in the Eastern Cape Province of South Africa. This study aimed to explore farmers’ perceptions of stock theft in the region. A mixed methods approach was used to collect data. 192 pre-tested questionnaires were collected from a sample of farmers in three districts in the province. The descriptive and chi-square test was used to test the associations between demographic profile statistically, knowledge of stock theft, reported stock theft cases, the economic impact of stock theft, and stock theft control. According to the findings, stock theft is significantly more likely to occur during the winter season (P < 0.05). About 94.8% of farmers are in the communal farming sector in the three districts visited. Furthermore, 81.2% of the respondents believe that the government needs to do more to combat the spread of stock theft. This study also revealed that most respondents (88.6%) agree that branding and tattooing should be made available to all registered farmers, while 53.1% believe that forensic deoxyribonucleic acid should not be used to control stock theft at crime scenes. This study highlights farmers’ perceptions and knowledge of stock theft to enable policymakers to develop targeted interventions and strategies. Policing strategy must be adaptive and technology-driven to fast-track detection, prevention, and reduction of stuck theft crime.

## 1. Introduction and background

An increase in stock theft numbers is expected, and this poses a significant threat to the food security of South Africa [1, 2]. According to recent statistics, stock theft is becoming a problem in South Africa and neighboring countries [3]. Stock theft statistics have been increasing in an exponential rate in the 2022/2023 financial year compared to the statistics of 2018/2019 financial year [4]. The numerous reports of stock theft cause financial loss to farmers, loss of good genetics, affecting job opportunities, thus affecting the food security of the country [5]. In addition, stock theft can have a severe negative impact on revenue because repeated stock theft can lead to market instability as stolen animal is a loss of for farmers [6].

The Eastern Cape Province (ECP) is characterised by a diverse agricultural landscape, with numerous small-scale farmers relying heavily on their livestock for income and subsistence [6]. The impact of stock theft on these farmers is far-reaching, as it not only results in financial losses but also undermines the region’s agricultural productivity and market competitiveness. Furthermore, the indirect consequences of stock theft, such as reduced market effectiveness and increased transaction costs, contribute to a complex web of challenges for farmers [7]. Direct and indirect impacts of stock theft also result in decreased market effectiveness and smaller agricultural networks, which reduce the availability of goods and raise prices and transaction costs [8].

Farming promotes and supports disadvantaged people’s livelihoods across the continent [9]. The ECP experiences high prevalences of stock theft amongst the other provinces in South Africa because ECP has so many borders that on crime in rural areas and stock theft [5, 10]. Among the most affected by stock theft areas are Qumbu, Tsolo, and Mthatha, which all fall under the OR Tambo district municipality [3]. With stock theft becoming a problem in ECP, cattle, goats, and sheep are the most vulnerable livestock animals to thieves because of their market value and easy trade in the black market [11]. Most ECP small-holder farmers consider doing rituals with livestock before they intend on selling them, especially goats, as they are the ones that people usually use to perform rituals.

The use of new technologies such as tracking device and radio frequency identification (RFID), and the development of necessary machines can be important tools in preventing stock theft. Globally, animal science and technology have been primarily responsible for increased livestock numbers and productivity; therefore, this technology must be implemented in conjunction with strategies to prevent stock theft or catch livestock thieves [12, 13]. In line with technological advancements and developments, the most used source is animal tracking, with the owner being able to track the movement of his herd on his phone/computer [11]. However, this technology’s economic and financial worth is still being debated in the small-holder farming system.

Despite the seriousness of the issue, there is a paucity of research focusing on farmers’ perceptions of stock theft in the ECP. Additionally, authorities can develop more effective strategies to stop stock theft, protect farmers’ livelihoods, and bring criminals to justice through learning about the perception and understanding of farmers. By filling this research gap, the study aims to shed a light on the specific dynamics and complexities of stock theft in the region, providing valuable insights for policymakers, law enforcement agencies, and farmer organizations to develop targeted interventions.

## 2. Materials and methods

### 2.1 Ethical consideration

The ethical clearance certificate was obtained and released by the University of Fort Hare Research Ethics Committee (UFHREC) in 2022 with the project number **JAJ031SNDZ01** before the procession of data collection, and the ethical clearance certificate is **REC-270710-028-RA Level 01**. Approval was obtained from each participant prior to the commencement of the interview. Participation in the survey was voluntary and participants were informed of their rights to withdraw from the survey at any stage.

### 2.2 Study area

**Fig 1:**
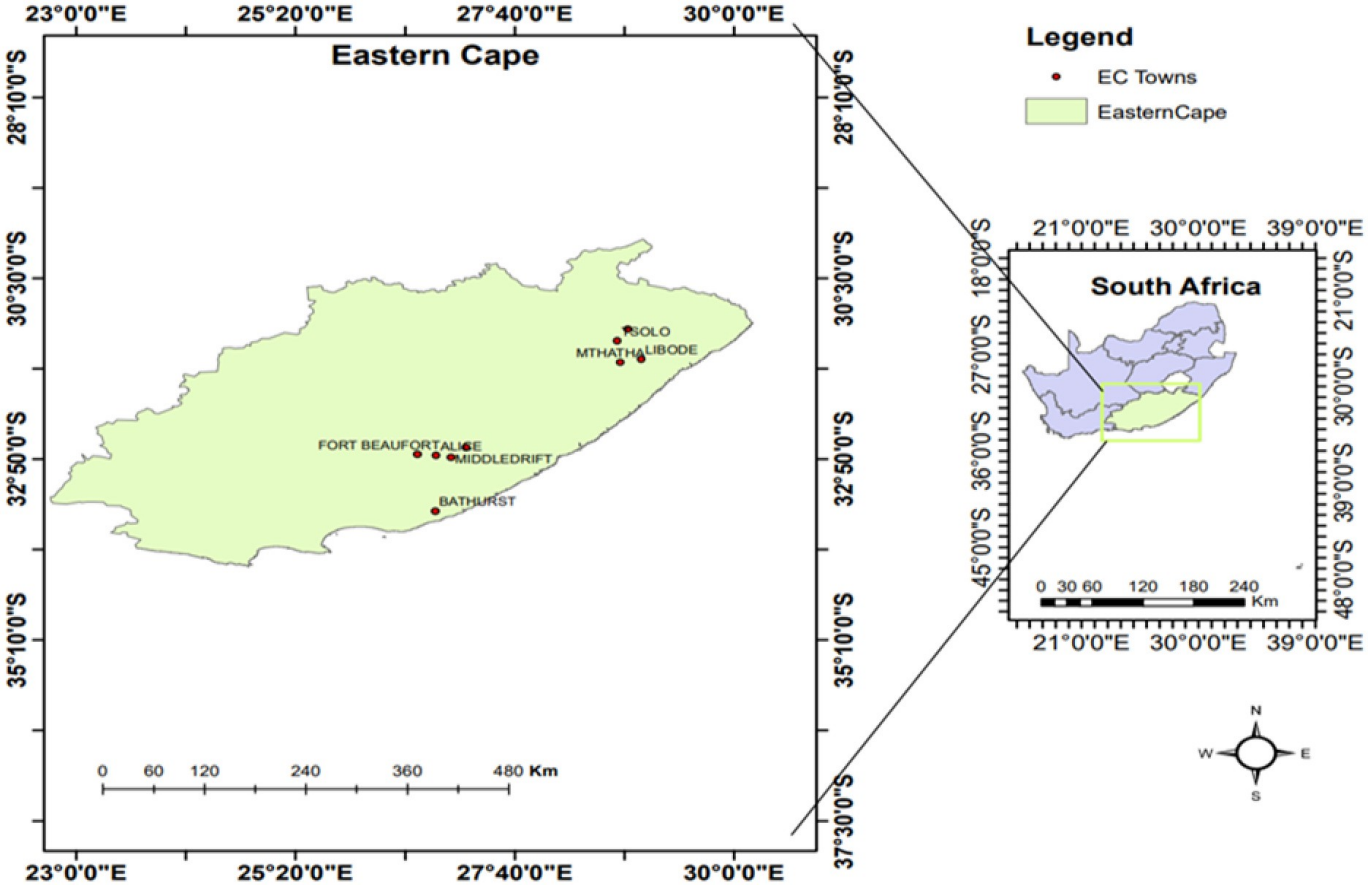
Distribution of study site.

The study was conducted in the ECP, mainly in 03 district municipalities: Amathole (Alice, Fort Beaufort, Middledrift, and Kieskammahoek), OR Tambo (Mthatha, Libode, Tsolo, and Qumbu), and Sarah Baartman (Bathurst and Makhanda) districts (Fig 1). The ECP share its border not only with the five provinces of South Africa but even with Lesotho. The reason for choosing these three district municipalities amongst the other municipalities is because, according to recent reports, it is where several cases of stock theft tend to happen via media reports [14]. The province makes up approximately 169 580 km^2^ (13.9%) of the country’s total land area, with so much significant unemployment rate in the last quarter of the year (December to march) and there was also a decline of 0.3% making the unemployment rate to be 39.7% [15].

### 2.3 Study population

Two hundred (200) communal and commercial farmers were targeted in three district municipalities to participate in this study. Communal farmers and commercial farmers were differentiated according to this manner: communal farmers typically involve multiple individuals or families working collectively on a piece of land, often owned or managed by a community or tribe while commercial farmers are large scale farmers who produce livestock in markets for big profit than communal farmers [16]. However, due to the unfinished answering and responding to the questions in the pre-tested questionnaire, our target changed from 200 to 192 collected questionnaires. Farmers participated voluntarily and it was a pre-tested questionnaire. For every household visited during data collection, we targeted the livestock owner or the person who looks after the livestock if the owner is unavailable.

### 2.4 Data collection methods

A structured pre-tested questionnaire was developed and pre-tested among Master of Science in Agriculture (Animal Science and Pasture Science) students in the Department of Livestock and Pasture Science, University of Fort Hare, South Africa. The mixed methods (both quantitative and qualitative) were used in this study. The pre-tested questionnaire was developed in English, but during data collection, it was translated into *isiXhosa*, to accommodate non-English speaking people. The mixed method approach allows researchers in agriculture and life sciences domain to pay careful attention to the ‘best’ approach to designing, implementing, analyzing, and integrating both qualitative (word) and quantitative (number) information and writing this in such a manner that offers better understandings and enhances its applicability and reproducibility [17]. The snowball probability was also used to reach out to other farmers in the area. The questionnaire was divided into four sections: (A) Demographic information; (B) Knowledge of stock theft and management systems; (C) The economic impacts of stock theft on farmers; (D) Control of stock theft and the government’s role in mitigating stock theft.

### 2.5 Statistical analysis

Data collected through questionnaires was then coded in Microsoft Excel to make the process of quantitative analysis of the data easier [18]. Descriptive statistical analysis was performed using IBM Statistical Package for the Social Sciences (SPSS), version 28.0.0.0 (190). Programmed random occurrence frequency was used to determine frequencies for demographic information and associations among variables of nominal data. Farmer’s perceptions and knowledge of stock theft in the ECP were assessed with a total of 32 questions. The questions that were correctly answered by most of the respondents successfully earned a point, and the ones that they failed to answer received a zero [15]. The Chi-square (X^2^) test was used for the associations among categorical variables. The study results were considered significant in a case where P ≤ 0.05.

## 3. Study findings and results

### 3.1 Demographic profile of the respondents

According to Table 1, 78.6% of the respondents were males, while 21.4% were females. Most respondents were between 46 and 66 (44.3%), and 2.1% were aged <25 years. Additionally, most of the respondents (93.8%) were black, while 6.2% accounted for the colored race. In terms of educational background, approximately 45% of the respondents had attained a secondary school level of education, while 21.4% had achieved a tertiary (post-secondary) level of education. Most respondents had over 30 years of experience of farming, and 20.8% had 11–20 years of farming experience. Furthermore, 39.1% of the respondents were unemployed, and 28.6% were employed.

**Table 1:** Demographic profile of respondents (n = 192).

Variable	Category	Frequency (n)	Percentage (%)
Gender	Male	151	78.6
	Female	41	21.4
Age in years	<25	4	2.1
	26–45	39	20.3
	46–66	85	44.3
	>66	64	33.3
Race	African	180	93.8
	Colored	12	6.2
Educational status	Primary	73	38
	Secondary	78	40.6
	Tertiary	41	21.4
Experience at farming	<10	44	22.9
	11–20	40	20.8
	21–30	50	26.0
	>30	58	30.3
Occupation	Employed	55	28.6
	Unemployed	75	39.1
	Retired/Pensioner	62	32.3

### 3.2 Distribution of variables related to the district, farm type, management system, and breed type

Most respondents (51.6%) were from Amathole, and 18.2% were from Sarah Baartman, as shown in Table 2. Most respondents (94.8%) were under the communal farm type, compared to 5.2% under the commercial farm type. Additionally, the majority of the respondents (87.5%) keep and raise their animals extensively, and 12.5% keep and raise their animals intensively. Furthermore, most respondents (88.5%) farm cattle, goats, and sheep, while 11.5% farm pigs and chickens only (Table 2).

**Table 2:** Distribution of variables related to district, farm type, management system, and type of breed.

Variable	Category	Frequency (n)	Percentage (%)
District	Amathole	99	51.6
	OR Tambo	58	30.2
	Sarah Baartman	35	18.2
Farm Type	Communal	182	94.8
	Commercial	10	5.2
Management System	Extensive	168	87.5
	Semi-extensive	24	12.5
	Intensive	0	0
Type of breed farmed	Cattle-Goat-Sheep	170	88.5
	Pig-Chicken	22	11.5
	Other	0	0

### 3.3 Association between variables and stock theft-related factors

According to Table 3, The is more likelihood of animal being stolen and there are high significant chances that after being stolen it will not be recovered (OR = 1.07–1.28; P<0.01). Additionally, there is less likelihood that stock theft occurs during all seasons but when animal is stolen there is significant that it will not be recovered (OR = 0.11–1.38; P<0.05) (Table 3).

**Table 3:** Association between variables and stock theft-related factors.

Variable	Level	Odds ratio	P-Values
Type of animal species farmed	Cattle-Sheep-Goat		
	Pig-Chicken		
	Donkey-Mule-Horse	1.07–1.28	0.001
Type of farm	Commercial		
	Communal	0.11–1.03	0.801
Management System	Extensive		
	Semi-extensive	2.10–2.43	0.0001
Level of stock theft	Low		
	Medium		
	High	1.33–2.01	0.0001
Livestock mostly exposed to stock theft	Cattle-Goat-Sheep		
	Pig-Chicken		
	Donkey-Mule-Horse	0.03–1.06	0.001
Season	Summer		
	Winter		
	Autumn		
	Spring	0.11–1.38	0.002

### 3.4 Response to the question—Is the government doing enough to prevent stock theft?

About 78.6% of males and 21.4% of females indicated that the government is not doing enough to prevent stock theft. Additionally, most Africans (93.8%) said that the government is not doing enough to prevent stock theft. Furthermore, there was a significant (P < 0.05) association between demographic variables and responses to the question: Is the government doing enough to prevent stock theft? (Table 4).

**Table 4:** Response to the question—Is the government doing enough to prevent stock theft? (n = 192).

Variable	Category	Frequency (n)	Percentage (%)	Is the government doing enough to fight- off stock theft?		*X* ^*2*^	Significance
				Yes	No		
Gender	Male	151	78.6	60	91		
	Female	41	21.4	9	32	57.019	0.0001
Age in years	<25	4	2.1	1	3		
	26–45	39	20.3	10	29		
	46–66	85	44.3	27	58		
	>66	64	33.3	13	51	49.158	0.001
Race	African	180	93.8	50	130		
	Colored	12	6.2	9	3	51.408	0.004
Educational status	Primary	73	38	20	53		
	Secondary	78	40.6	50	28		
	Tertiary	41	21.4	38	3	50.817	0.002
Experience at farming	<10	44	22.9	25	19		
	11–20	40	20.8	15	25		
	21–30	50	26.0	19	31		
	>30	58	30.3	11	47	39.426	0.01
Occupation	Employed	55	28.6	40	15		
	Unemployed	75	39.1	25	50		
	Retired/Pensioner	62	32.3	18	44	46.294	0.002

### 3.5 Perception and attitudes toward stock theft and stock theft control measures

Many respondents (94,3%) believe the season has an effect on stock theft, while other respondents (5.7%) do not believe stock theft season has an effect on the season (Table 5). Furthermore, 81.2% of respondents believe the government needs to do more to prevent stock theft. Additionally, 78.1% of respondents said no steps were taken to curb the spread of stock theft. Farmers’ perceptions and responses to question seasonality, prevalence, mitigating action and government effort to curbing stock theft were statistically significant (P <0.05) (Table 5).

**Table 5:** Farmer’s perception and attitudes towards stock theft.

Questions	n	A		B		C		Total (%)		*X* ^*2*^	Significance
		Yes (%)	No	Yes (%)	No	Yes	No	Yes	No		
Have you ever reported stock theft?	192	80 (41.7)	19 (9.9)	41 (21.4)	17 (8.9)	23 (11.9)	12 (6.3)	75	25	42.019	0.001
Does season have an impact on stock theft	192	98 (51.0)	1(0.5)	51 (26.6)	7 (3.6)	32 (16.7)	3 (1.6)	94.3	5.7	51.817	0.001
As a community, is there any step you have taken to curb the spread of stock theft?	192	8 (4.2)	91 (47.4)	31 (16.1)	27 (14.1)	3 (1.6)	32 (16.6)	21.9	78.1	62.081	0.001
Number of stock theft incidence	192	62 (32.3)	37 (19.3)	22 (11.5)	36 (18.8)	34 (17.7)	1 (0.5)	61.4	38.6	42.960	0.0001
Is the government doing enough to prevent stock theft?	192	22 (11.5)	77 (40.1)	4 (2.1)	54 (28.1)	10 (5.2)	25 (13.0)	18.8	81.2	67.019	0.0001

Statistically significant at P≤ 0.05: A: Raymond, B: OR Tambo, C: Sarah Baartman

Most respondents (77.1%) believe that implementing a lifetime prison sentence will be enough. Additionally, majority of the respondents (88.6%) said that branding and tattooing should be made available to all registered farmers as it is a suitable identification method. Majority of respondents (90.6%) of respondents acknowledged that government should compensate for stolen livestock. Most respondents do not believe forensic DNA should be used to control stock theft at crime scenes (53.1). Additionally, 68.8% of the respondents said that using the snap animal application identification for cattle identification will not work (Table 6). Table 7 shows association between demographic profile and significance where P< 0, 05 with factors such as knowledge of stock theft, report case of stock theft, the economic impact of stock theft, and control of stock theft.

**Table 6:** Famers response and attitudes towards stock theft control measures in three different districts.

Questions	n	A		B		C		Total (%)		*X* ^*2*^	Significance
		Yes (%)	No	Yes (%)	No	Yes (%)	No	Yes	No		
Will the implementation of a lifetime prison sentence be enough?	192	69 (35.9)	30 (15.6)	50 (26.0)	8 (4.2)	29 (15.1)	6 (3.1)	77.1	22.9	63.319	0.0001
Do you approve government should compensate people for stolen livestock?	192	91 (47.4)	8 (4.2)	52 (27.1)	6 (3.1)	31 (16.1)	4 (2.1)	90.6	9.4	75.019	0.0001
Government should provide GPS tracking devices to farmers at an affordable price	192	18 (9.4)	81 (42.2)	17 (8.9)	41 (21.4)	15 (7.8)	20 (10.4)	26	76	43.905	0.001
Branding and tattooing should be made available to all registered farmers	192	90 (46.9)	9 (4.7)	51 (26.6)	7 (3.6)	29 (15.1)	6 (3.1)	88.6	11.4	55.913	0.001
Do you believe forensic DNA should be used to control stock theft at crime scenes?	192	70 (36.5)	29 (15.1)	15 (7.8)	43 (22.4)	5 (2.6)	30 (15.6)	46.9	53.1	60.241	0.001
Snap animal app for cattle identification	192	30 (15.6)	69 (35.9)	20 (10.4)	38 (19.8)	10 (5.2)	25 (13.1)	31.2	68.8	53.629	0.001
The use of drone technology to reduce stock theft	192	59 (30.7)	40 (20.8)	36 (18.8)	22 (11.5)	20 (10.4)	15 (7.8)	59.9	40.1	41.216	0.001

Significant at P < 0.05; A: Raymond, B: OR Tambo, C: Sarah Baartman

**Table 7:** Association between demographic profile, knowledge of stock theft, report of stock theft, the economic impact of stock theft, and stock theft control.

Demographic profile	Knowledge of stock theft	Report case of stock theft	The economic impact of stock theft	Stock theft control
**Gender**	0.001**	0.002**	0.001**	0.001**
**Age**	0.05**	0.001**	0.001**	0.001**
**Race**	0.01**	0.001**	0.0001***	0.001**
**Educational status**	0.42^NS^	0.001**	0.001**	0.001**
**Farm experience**	0.001**	0.002**	0.001**	0.001**
**Occupation**	0.84^NS^	0.023*	0.001**	0.001**

Significant at ***P < 0.0001, **P < 0.01, and *P < 0.05 and NS not significant at P >0.0

## 4. Discussions

The study results showed that most respondents were male (78.1%), which could be attributed to the physical demands of livestock management that require manpower for animal care and treatment. These findings align with previous studies conducted in Africa and Asia [15, 19, 20]. However, it is essential to acknowledge that women play a vital role in agricultural production, often regarded as the backbone of farming communities, contributing significantly to food security and rural development [21].

In terms of age distribution, 44.3% of the respondents fell within the age group of 46–66. These findings are consistent with previous studies focusing on communal farmer-owners who rely solely on farming as their source of income as reported [22, 23]. Economic factors, such as young people leaving communal areas for better opportunities in urban areas, often leave older individuals as communal farmers [24].

Regarding education, a significant proportion of farmers in the current study (40.6%) completed secondary school. These findings are in line with recent studies by indicating that many farmers have no basic education or education up to the primary level [20, 22]. Furthermore, a substantial number of respondents (30.3%) had over 30 years of farming experience. This observation is consistent with studies conducted in the Eastern Cape Province (ECP) and Limpopo Province as reported by recent studies [22, 25]. It is also noteworthy that older farmers often rear livestock passed down from their forefathers. Additionally, a significant percentage of the respondents (39.1%) were unemployed, representing an increase compared to previous studies [9, 26]. It is well known that amongst the nine provinces of the country, ECP is the leading province in terms of unemployment rate. This drivers individuals to turn into farming for income and household subsistence [27].

Most farmers in the ECP practice communal farming, while commercial farming is less common. The prevalence of impoverished rural communities, especially among communal farmers with limited income opportunities, shapes this trend [28]. The study revealed that communal farmers in the three visited districts represented the majority (94.8%), using traditional technology systems. These findings are supported by a study done in the Eastern Cape and Free State Provinces, where small-scale farmers rely on limited income sources, such as pensions, which are often insufficient to support their livelihoods [29]. Given the communal nature of farming, most respondents (87.5%) used an extensive management system, which improved soil fertility and reduced costs compared to frequent feed purchases [30]. Furthermore, the growth rate differs between extensive and semi-intensive systems, because animals in the farmer have free access to pasture, while the latter restricts grazing.

The study also revealed that the most farmed animals (85.5%) were cattle-goats-sheep (ruminants). This preference for ruminant animals can be attributed to the multiple byproducts they produce, such as mohair, wool, horns, and hooves, compared to non-ruminant animals [31]. Moreover, ruminants, particularly cattle, can be used for plowing, as modern machinery like tractors can negatively affect soil components due to their weight. Respondents agreed that the high level of stock theft in their respective districts could be attributed to the ECP’s high unemployment rate. Among the nine provinces of South Africa, the ECP has the highest number of livestock. Furthermore, stock theft is a widespread issue, extending beyond South Africa’s borders to neighbouring countries and even European countries, including Russia, as evidenced by previous research [32–34]. However, the scale of theft in European countries may vary due to farm intensification.

Farmers believe that livestock are more susceptible to theft during the winter months, due to the shorter daylight hours and extended periods of darkness, making them more vulnerable to stock theft. The distance to grazing camps is long and traditional events such as *imigdi*, *imbeleko*, *lobola*, funerals, and graduation ceremonies contribute to the increase of livestock theft because people tend to for a purchase of cheap livestock between these sales, there is no need of certificate of ownership. Offenders take advantage of these factors by stealing livestock and selling it for these events or slaughtering it for sale to butcheries. Similar findings have been reported in studies conducted in Limpopo and Free State Province [35, 36].

Continued stock theft poses risks to agricultural production, food security, and the pricing of meat and livestock byproducts. It also increases the potential outbreak of contagious diseases, such as foot and mouth, as stolen livestock from South Africa is often smuggled to Lesotho and vice versa [37, 38].

Farmers in the current study expressed dissatisfaction with the government’s efforts to combat stock theft. This expression could be linked to the continuous theft of livestock in these communities, as stock theft is a severe crime that impacts not only farmers but the agricultural industry as a whole and the economy in general. To effectively address it, stakeholders like farmers, South African Police Service (SAPS), the Department of Rural Development and Agrarian Reform (DRDAR) must adopt an extensive and integrated strategy [39, 40]. Additionally, by acknowledging the syndicated nature of stock theft and working together these stakeholders can disrupt criminal networks and protect the livelihoods of farmers [41].

Most respondents (77.1%) believed that implementing lifetime prison sentence would be sufficient to deter stock thieves, as current sentences are perceived as lenient. These findings are supported by a recent study that emphasizes the need for the government to review the crimes committed and consider increasing penalties, otherwise many aspects of the country could be jeopardized [3]. Harsh prison sentences would also contribute to a better environment and the well-being of farmers, particularly considering that they are often elderly and susceptible to mental distress when their animals are stolen [22, 33].

Furthermore, most farmers in the current study believed that branding and tattooing of livestock should be made available to all registered farmers (88.6%). This could be attributed to the fact that branding and tattooing are not fully distributed widely across South Africa by the DRDAR to farmers as it is one of the important identification methods in combating stock theft. The lack of financial resources may contribute to an increased vulnerability to stock theft, as these farmers cannot afford to hire herders to look after their livestock and elderly people do receive social grants, livestock provides sustainability for rural old people and others [37]. Consequently, farmers have resorted to building camps for their livestock using wire structures to facilitate control and management [42, 43].

## 5. Conclusion and recommendations

The study highlights the perception of stock theft in the ECP, South Africa. However, the province struggles to control stock theft due to poor infrastructure effectively. The study also revealed that farmers heavily rely on livestock as their primary source of income. Respondents expressed dissatisfaction with the government’s and SAPS’ efforts to combat stock theft. Despite the challenges faced, farmers demonstrated a strong awareness of preventive measures and the utilization of various strategies to protect their livestock, such as improved fencing, guard dogs, and community watch programs. However, limited resources and financial constraints often hampered the implementation of these measures, particularly among small-scale farmers. Farmers needed enhanced collaboration between farmers, local communities, and law enforcement agencies to combat stock theft effectively. Policing strategy must be adaptive and technology-driven to fast-track detection, prevention, and reduction of stuck theft crime. Further research is needed to explore the effectiveness of community-based initiatives and the role of technology in combating stock theft in this region.

## Supporting information

S1 Data (XLSX)

S1 Questionnaire (DOCX)
