# Supplementary material for: Farmers’ perceptions on stock theft in some districts of the Eastern Cape Province, South Africa
Source: PLoS One. 2024 Sep 27;19(9):e0310881. doi: 10.1371/journal.pone.0310881 (PMC11433143; doi:10.1371/journal.pone.0310881)
Supplement: S1 Questionnaire — (DOCX) [file pone.0310881.s002.docx]

**Please Note:**

***This questionnaire is designed to assess farmer's perception and prevalence of stock theft in communal and commercial farmers in Eastern Cape Province, South Africa. This questionnaire is being carried out solely for academic purposes, and no information gathered will be used against any farmer. Your response and cooperation will be immensely appreciated.***

**Farmer’s information**

| Name of a farmer: ……………………… | Municipality: ……………………. |
| --- | --- |
| Name of a Town : ……………….. | Date: …………………………. |

**Section A: Demographic information of a farmer**

1.1 Gender : Male Female

1.2 Age : <25 25 – 45
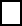
 46 – 66
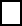
 >66
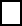


1.3 Race : African
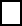
 Colored
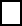
 White
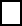
 Indian
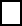


1.4 Educational level: Primary
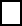
 Secondary
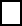
 Tertiary
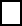


1.5 Experience at farming: < 10
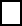
 11 – 20
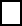
 21 - 30
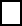
 > 30
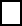


1.6 Occupation : Employed
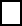
 Unemployed
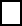
 Retired/ Pensioner
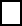


1.7 Number of Livestock: Cattle ……….

Goat…………

Sheep……….

Pig …………

Chicken……..

If other, please specify the name of livestock and number ………………………….

**Section B: Knowledge of stock theft and management system**

|  | **Item** | **Yes** | **No** |
| --- | --- | --- | --- |
| 1 | Farm Type   1. Commercial |  |  |
|  | (b) Communal |  |  |
| 2 | Management System |  |  |
|  | 1. Extensive System |  |  |
|  | 1. Semi-Extensive System |  |  |
|  | c) Intensive System |  |  |
| 3 | Type of breed farmed |  |  |
|  | 1. Cattle-Goat-Sheep |  |  |
|  | 1. Pig-Chicken |  |  |
|  | c) Donkey-Horse-Mule |  |  |
| 4 | Have you experienced stock theft before? |  |  |
| 5 | Level of stock theft in the area |  |  |
|  | 1. Low |  |  |
|  | 1. Medium |  |  |
|  | (c) High |  |  |
| 6 | Livestock mostly exposed to stock theft? |  |  |
|  | (a) Cattle-Goat-Sheep |  |  |
|  | (b) Pig-Chicken |  |  |
|  | 1. Donkey-Horse-Mule |  |  |
| 7 | A season where stock theft is likely to occur the most? |  |  |
|  | 1. Summer 2. Winter 3. Autumn   (d) Spring | | |
| 8 | Have you reported the cases of stock theft to the SAPS? |  |  |
| 9 | Possible reasons for not reporting stock theft? |  |  |
|  | 1. Too far from the SAPS |  |  |
|  | 1. Low service delivery from the SAPS regarding cases of stock theft |  |  |
|  | (c)…………………………………………………………………………………………………………………………….. | | |
| 10 | As a community/farmer in the area, are any steps you have done to curb the spread of stock? ……………………………………………………………………………………….  ……………………………………………………………………………………….  ………………………………………………………………………………………. | | |
|  |  |  |  |

**Section C: The economic impacts of stock theft in farmers**

|  | **ITEM** | **Yes** | **No** |
| --- | --- | --- | --- |
| 1 | Number of stock theft incidents |  |  |
|  | 1. Livestock Stolen ………………… | | |
|  | (b) Livestock Recovered …………….. | | |
| 2 | How often do you count your animals? |  |  |
|  | 1. Once a week |  |  |
|  | 1. Twice a week |  |  |
|  | 1. All week |  |  |
| 3 | Is the government doing enough to fight-off stock theft? |  |  |
| 4 | Reasons for livestock farming |  |  |
|  | 1. Food for household |  |  |
|  | (b) Income and wealth creation |  |  |
|  | c) …………………………………………………………………………………. | | |
| 5 | Estimation of costs lost due to stock theft? |  |  |
|  | 1. < R20 000 |  |  |
|  | 1. > R20 000 |  |  |
| 6 | What are contributing factors to the increased stock theft rate? |  |  |
|  | 1. Unemployment Rate |  |  |
|  | 1. Youth exposed more to drugs |  |  |
|  | 1. Less conviction rates / lawlessness |  |  |
|  | d) …………………………………………………………………………………… | | |

**D. Control of Stock theft, the government needs to do the following?**

|  | **Items** | **Yes** | **No** |
| --- | --- | --- | --- |
| **1** | Will implementation of a harsh prison sentence for someone found stealing livestock? |  |  |
| **2** | Do you approve/ believe that government should compensate those affected by stock theft? |  |  |
| **3** | Government should provide GPS tracking devices to farmers at an affordable price |  |  |
| **4** | Branding and tattooing should be made available to all registered farmers with livestock |  |  |
| **5** | As a farmer, do you believe forensic DNA should be used as the main control of stock theft at crime scenes? |  |  |
| **6** | Snap animal app for cattle identification |  |  |
| **7** | The use of drone technology to reduce livestock theft |  |  |
